# Interpersonal violence among in-school adolescents in sub-Saharan Africa: Assessing the prevalence and predictors from the Global School-based health survey

**DOI:** 10.1016/j.ssmph.2021.100929

**Published:** 2021-10-01

**Authors:** Richard Gyan Aboagye, Abdul-Aziz Seidu, Collins Adu, Abdul Cadri, Dickson Okoree Mireku, Bright Opoku Ahinkorah

**Affiliations:** a Department of Family and Community Health, School of Public Health, University of Health and Allied Sciences, Ho, Ghana; b Department of Population and Health, University of Cape Coast, Cape Coast, Ghana; c College of Public Health, Medical and Veterinary Services, James Cook University, Australia; d Department of Estate Management, Takoradi Technical University, P.O. Box, 257, Takoradi, Ghana; e Department of Health Promotion, Education and Disability Studies, Kwame Nkrumah University of Science and Technology, Kumasi, Ghana; f Department of Family Medicine, Faculty of Medicine, McGill University, Montreal, Quebec, Canada; g Directorate of Academic Planning and Quality Assurance, University of Cape Coast, Ghana; h School of Public Health, Faculty of Health, University of Technology Sydney, Sydney, Australia

**Keywords:** Interpersonal violence, In-school adolescents, sub-Saharan Africa, Global health

## Abstract

Interpersonal violence in adolescents has over the years grown into a serious public health problem that merits a robust intervention. This study, therefore, assessed the prevalence and predictors of interpersonal violence among in-school adolescents in sub-Saharan Africa (SSA). The study involved a cross-sectional analysis of data from the Global School-based Health Survey conducted between 2012 and 2017 from eight sub-Saharan African countries. A total of 14,967 in-school adolescents aged 10–19 years were included in the pooled analysis. A multivariable binomial logistic regression was used to determine the predictors of interpersonal violence using the adjusted odds ratio (aOR) with their respective 95% confidence intervals (CIs). The overall prevalence of interpersonal violence among in-school adolescents in SSA was 53.7%. The odds of interpersonal violence were higher among adolescents who were bullied (aOR = 2.52, 95% CI = 2.23–2.85), had an injury (aOR = 2.42, 95% CI = 2.15–2.72), had suicidal attempts (aOR = 1.40, 95% CI = 1.16–1.70), were truant (aOR = 1.51, 95% CI = 1.33–1.72), used alcohol (aOR = 1.49, 95% CI = 1.06–2.11), and used tobacco (aOR = 1.46, 95% CI = 1.23–1.74). In-school adolescents with peer support, parents or guardians bonding, those whose parents or guardians respected their privacy, and those aged 15 years or older were less likely to experience interpersonal violence. These factors provide education directors and school heads/teachers with relevant information to guide the design of specific interventions such as parent-teacher meetings and programs, peer educator network system, face-to-face counseling sessions, Rational Emotive Behavioural Education (REBE) and substance use cessation therapy to prevent interpersonal violence, particularly physical fights and attacks in school settings. Also, students should be sensitized on the negative effects of interpersonal violence and those who have been exposed to it should be counselled. School rules should be strengthened and appropriate punishment given to students who engage in violence baheviours in schools in order to deter others from engaging in them.

## Highlights

• The overall prevalence of interpersonal violence among in-school adolescents in sub-Saharan Africa was 53.7%.
• Several socio-demographic, psychosocial and environmental factors are associated with interpersonal violence among in-school adolescents in SSA.
• School rules should be strengthened and appropriate punishment given to students who engage in violence baheviours in schools in order to deter others from engaging in them.
• The design and implementation of specific interventions such as parent-teacher meetings or programs to help parents improve their relationships with in-school adolescents can help reduce inter-personal violence in schools.

## Authors statement

RGA and AS conceived the study. BOA and RGA designed and performed the analysis. RGA, AS, CA, AC, DOM and BOA designed first draft of the manuscript. RGA, AS, CA, AC, DOM, and BOA revised the manuscript for intellectual content and gave consent for the version to be published. All authors have read and approved final manuscript and agreed to be accountable for all aspects of the work.

## 1 Introduction

Interpersonal violence, which involves a deliberate use of power or physical force against an individual or a limited group of people, is the 19th most common cause of death globally, accounting for about 410,000 deaths annually (Fazel et al., 2018; Mercy et al., 2017; Wang et al., 2016). This violence may take different forms: physical, sexual, or psychological, and it may involve deprivation or neglect (Mercy et al., 2017). Adolescence is a critical stage of life with rapid changes in physical, psychological, and cognitive structures, as well as experience of stress and experimentation (Miller et al., 2019; Perry et al., 2020; Roman & Frantz, 2013). These changes predispose adolescents to risky and unhealthy behaviours such as substance use, school dropout, high-risk sexual behaviours, and early pregnancy (Miller et al., 2019). Interestingly, interpersonal violence plays a significant role in the likelihood of an adolescent engaging in such behaviours by presenting up to two folds likelihood (Roman & Frantz, 2013; Silverman et al., 2011).

Interpersonal violence in adolescents has over the years grown into a serious public health problem that merits a robust intervention. It is reported that every 7 minute, somewhere in the world, an adolescent dies from an act of violence (United Nations Children's Fund, 2017). Also, in 2015 alone, about 82,000 adolescent deaths were attributed to violence, with the highest rate in adolescents aged 15–19 (United Nations Children's Fund, 2017). Interpersonal violence was the second leading cause of death in this age group in 2016 (World Health Organization [WHO], 2017). Interpersonal violence among adolescents leads to physical injuries in the form of bruises, wounds, fractures, broken teeth, disability, and head injuries (Buchanan, 2014; Norton & Kobusingye, 2013). It also has been linked with infectious diseases, reproductive health problems, and mental health problems such as mood disorders, anxiety, and suicidal ideations (Devrieset al., 2011; Fisher et al., 2012; Machtinger et al., 2012).

Interpersonal violence among adolescents is prevalent in most parts of the world and sub-Saharan Africa (SSA) is no exception. In SSA, the prevalence rate of interpersonal violence among in-school adolescents ranges from 26% in Nigeria (Ngugen et al., 2021) to 55.7% in Ghana (Aboagye et al., 2021). Interpersonal violence among adolescents has its associated factors. These factors have been reported by studies in SSA which include experience of hunger, lower socioeconomic status, age, living in a rural area, male sex, lack of parental or guardian supervision, substance use including alcohol, suicide attempt, injury, sedentary behaviour, psychological distress, truancy, and bullying victimization (Aboagye et al., 2021; Beyene et al., 2019; Acquah et al., 2014a, 2014b; Lozano et al., 2012; Norman et al., 2012).

With the recent public health shift towards prevention model and, delineating risk factors (WHO, 2014) for violence, considering the growing recognition of the detrimental effects of interpersonal violence, one may expect policies and interventions at the sub-regional level. Even though there have been some studies on interpersonal violence among adolescents in SSA, it remains difficult to compare the findings across these studies, as they differ in terms of measurement and sampling strategies, age groups interviewed and study objectives. Also, the huge differences in the socio-cultural and ecological make-up and context existing among sub-Saharan African countries that make comparison of studies from the region difficult, therefore, calling for a multi-country analysis with the use of uniform measures. The present study sought to fill this gap in literature. This study used data from the Global School-based Health Survey (GSHS) to assess the prevalence and risk factors of interpersonal violence among in-school adolescents in SSA. The findings of this study could be a stepping stone for designing and implementing policies and interventions that will prevent school-based interpersonal violence in SSA.

It is critical to apply theories and models to explain interpersonal violence among in-school adolescents for the purposes of this study. Interpersonal violence, according to the ecological model, is the result of interactions between various factors at four levels: the individual, the relationship, the community, and the society (WHO, 2021). The individual level factors include the socio-demographic characteristics of individuals that influence their behaviours and increase interpersonal violence victimization. These include victimization during childhood, psychological and personality factors, alcohol and/or substance abuse and experience of interpersonal violence in the past. The relationship factors focus on how interactions between individuals and family members, friends, intimidate partners, and peers increase their susceptibility to interpersonal violence. The community level factors are the contextual factors that predispose individuals to interpersonal violence such as unemployment, population density, and mobility. Societal level factors include the economic and social policies that maintain socioeconomic inequalities between people, and the social and cultural norms that support interpersonal violence (WHO, 2021). It is worth noting that human ecology is concerned with “the link between the population's environmental and demographic features, as well as the impact of these two broad groups of factors on human behavior” (McBride & McCoy, 1981. P.284). The ecological model examines a child's growth and behaviour within the context of the child's immediate environment (family, classmates, and school), as well as the larger social environment (society, culture, and community) (Kumpfer & Turner, 1990). Previous research has found that teenage ecological environments might either prevent or expose them to interpersonal violence (Moon et al., 2010). Only a few research in SSA have employed a theoretical model to support their findings on interpersonal violence among teenagers (Aboagye et al., 2021). Based on the tenets of the model, we hypothesized that socio-demographic, psychological, and environmental factors are linked with interpersonal violence among in-school adolescents in SSA.

### 1.1 Methods

#### 1.1.1 Data source and study design

This study involved a cross-sectional analysis of data from the GSHS conducted between 2012 and 2017 from eight sub-Saharan African countries. The data were pooled from Benin (2016), Ghana (2012), Liberia (2017), Mauritius (2017), Mozambique (2015), Namibia (2013), Seychelles (2015), and Tanzania (2014). The GSHS is a study conducted among in-school adolescents from countries with partnership with the WHO, Center for Disease Control and Prevention (CDC), and Middle Tennessee State University (MTSU). The surveys collect data on several adolescent health risks and protective factors using a structured questionnaire. Data collected during the survey include violence, unintentional injury, dietary behaviours, hygiene, mental health, physical activity, substance use, and sexual behaviours. The questionnaire used in this study has previously been published elsewhere (See Table S1). The dataset to the various surveys were accessed on April 1, 2021 and are freely available online. The links to the various datasets has been provided as a supplementary file 1 (Table S1).

#### 1.1.2 Sampling method and sample size

The GSHS utilised a two-stage cluster sampling technique in recruiting the study schools and students for the survey. During the first stage, study schools were selected with probability proportional to the school's enrolment size. Later, classes within chosen schools were randomly selected and all the students in the sampled classrooms were eligible to participate in the survey. The survey included students who were aged 10–19 years (period of adolescence), present at school on the day of data collection, and showed evidence of written informed consent (those aged 18 years and above), and written parental or guardian consent form and child assent form (those between 10 and 17 years). The sampling technique used ensured that every eligible student had an equal chance of being selected for inclusion in the study. Numerical weights were applied to each student record to enable the generalization of results to in-school adolescents. In the present study, a total of 14,967 in-school adolescents were included in the final analysis. Of this, the sample from each country was Benin (1671), Ghana (2214), Liberia (1167), Mauritius (1,995), Mozambique (1,033), Namibia (2,860), Seychelles (1,572), and Tanzania (2,455). The “Strengthening the Reporting of Observational Studies in Epidemiology” (STROBE) statement was used in writing the manuscript (Von Elm et al., 2014).

### 1.2 Study variables

#### 1.2.1 Outcome variable

The outcome variable in the present study is interpersonal violence. This variable was measured using two variables (physical fighting and physical attack). With physical fighting, the students were asked *“During the past* 12 months*, how many times were you in a physical fight?”.* For physical attack, the respondents were asked *“During the past* 12 months*, how many times were you physically attacked?”.* The responses in each of the two variables were *“1=0 times; 2 = 1 time; 3 = 2 or 3 times; 4=4 or 5 times; 5=6 or 7 times; 6=8 or 9 times; 7=10 or 11 times; and 8=12 or more times”.* The response options were further recoded into “No [0 times]” and “Yes [1 time to 12 or more times]”. The recoded responses in each of the variables were used to create an index variable called 'interpersonal violence'. The students who responded “No” after the recoding in both the physical fighting and physical attack were categorised as “not experienced interpersonal violence [No]” whilst those who responded at least “Yes” in any of the variables were grouped as “experienced interpersonal violence [Yes]”. Previous studies using the GSHS datasets have used the same categorisation in assessing interpersonal violence among in-school adolescents (Aboagye et al., 2021; Pengpid and Peltzer, 2020; Senanayake et al., 2019).

#### 1.2.2 Explanatory variables

We considered twenty-one variables as explanatory variables in the study. These variables were selected based on their availability in the GSHS datasets as well as their significant association with interpersonal violence (Aboagye et al., 2021; Pengpid & Peltzer, 2019; Senanayake et al., 2019; Rudatsikira et al., 2007, 2008). The ecological model of interpersonal violence, which is the model that guided this study also influenced the selection of the explanatory variables. The variables included age (years), sex, hunger, anxiety, loneliness, injury, bullying, current alcohol consumption, current tobacco use, current cigarette smoking, current marijuana use, suicidal ideation, suicidal plan, suicidal attempt, peer support, close friends, truancy, parental/guardian supervision, parental/guardian connectedness, parental/guardian bonding, and parental/guardian respect for privacy. A detailed description of the variables including the questions, response options, and coding can be found in the supplementary file attached (Table S2).

### 1.3 Statistical analyses

**Fig. 1:**
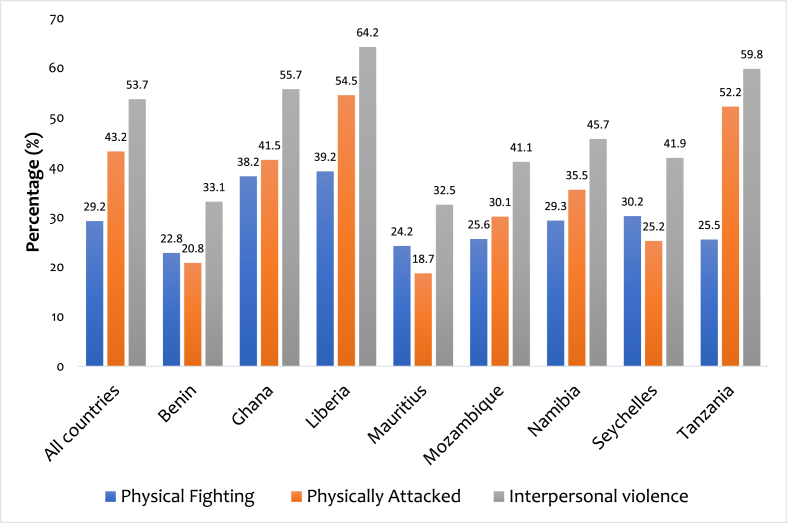
Prevalence of interpersonal violence among in-school adolescents.

We analysed the data using Stata software version 16.0 (Stata Corporation, College Station, TX, USA). The analysis was carried out in three stages. Firstly, percentages were used to present the results of the prevalence of physical fighting, physical attack, and interpersonal violence among in-school adolescents (Fig. 1). At the second stage, the Pearson chi-square test of independence was employed to examine the association between the explanatory variables and physical fighting, physical attack, and interpersonal violence (Table 1). A multivariable binomial logistic regression was later used to determine the association between the explanatory variables and physical fighting, physical attack, and interpersonal violence. All the explanatory variables that were statistically significant in any of the outcome variables during the chi-square test were placed in the regression model. The results of the regression analysis were presented in a tabular form using the adjusted odds ratio (aOR) with their respective 95% confidence intervals (CIs). All frequency distributions were weighted while the survey command (svy) in Stata was used to adjust for the complex sampling design of the data.

**Table 1:** Bivariate analysis of proportions of interpersonal violence among in-school adolescents in SSA.

Variable	Weighted N	Weighted %	Physical fighting		Physically attacked		Interpersonal violence	
			Yes (%)	P-value	Yes (%)	P-value	Yes (%)	P-value
**Age**				<0.001		0.008		<0.001
14 years or younger	6082	40.6	35.2		47.6		60.1	
15 years or older	8885	59.4	25.1		40.2		49.3	
**Sex**				<0.001		<0.001		<0.001
Female	6955	46.5	28.6		43.4		53.6	
Male	8012	53.5	29.7		43.1		53.7	
**Felt hungry**				<0.001		<0.001		<0.001
No	13,550	90.5	28.8		43.0		53.4	
Yes	1417	9.5	33.1		45.6		53.9	
**Injury**				<0.001		<0.001		<0.001
No	8190	54.7	17.5		33.5		42.0	
Yes	6777	45.3	43.3		54.9		67.7	
**Bullied**				<0.001		<0.001		<0.001
No	9905	66.2	20.2		36.5		45.3	
Yes	5062	33.8	46.8		56.3		70.1	
**Anxiety**				<0.001		<0.001		<0.001
No	13,436	90.4	28.2		42.3		52.8	
Yes	1431	9.6	38.2		51.4		61.8	
**Felt lonely**				<0.001		<0.001		<0.001
No	13,489	90.1	28.4		42.5		53.0	
Yes	1478	9.9	36.7		49.3		59.7	
**Suicide ideation**				<0.001		<0.001		<0.001
No	12,918	86.3	27.3		41.9		52.1	
Yes	2049	13.7	40.9		51.7		63.2	
**Suicide plan**				<0.001		<0.001		<0.001
No	12,970	86.7	27.7		42.2		52.5	
Yes	1997	13.3	39.0		49.6		61.1	
**Suicide attempt**				<0.001		<0.001		<0.001
No	12,916	86.3	26.6		41.2		51.5	
Yes	2051	13.7	45.2		55.9		67.1	
**Current alcohol use**				<0.001		0.101		<0.001
No	13,191	88.1	27.5		43.2		53.2	
Yes	1776	11.9	41.8		43.1		57.4	
**Current cigarette smoking**				<0.001		<0.001		<0.001
No	14,423	96.4	27.9		42.6		52.9	
Yes	544	3.6	64.1		59.2		73.6	
**Current marijuana use**				<0.001		<0.001		<0.001
No	14,684	98.1	28.5		42.8		53.1	
Yes	283	1.9	67.0		66.1		83.2	
**Current tobacco use**				<0.001		<0.001		<0.001
No	14,362	96.0	27.7		42.3		52.6	
Yes	605	4.0	65.1		65.8		78.0	
**Close friends**				0.129		0.608		0.883
No	1385	9.3	25.0		38.0		49.7	
Yes	13,582	90.7	29.6		43.7		54.1	
**Truancy**				<0.001		<0.001		<0.001
No	10,825	72.3	24.3		39.7		49.3	
Yes	4142	27.7	41.9		52.4		65.1	
**Peer support**				<0.001		0.046		<0.001
No	9818	65.6	32.1		44.0		55.8	
Yes	5149	34.4	23.7		41.8		49.7	
**Parent or guardian supervision**				0.560		<0.001		0.022
No	7482	50.0	30.6		41.6		53.0	
Yes	7485	50.0	27.8		44.9		54.4	
**Parent or guardian connectedness**				<0.001		<0.001		<0.001
No	8965	59.9	31.1		44.1		55.2	
Yes	6002	40.1	26.3		41.8		51.4	
**Parent or guardian bonding**				<0.001		<0.001		<0.001
No	9141	61.1	31.7		43.9		55.4	
Yes	5826	38.9	25.3		42.1		50.9	
**Parent or guardian respect for privacy**				<0.001		<0.001		<0.001
No	4199	28.1	36.1		48.0		60.5	
Yes	10,768	71.9	26.5		41.3		51.0	

Note: P-values were generated from the chi-square test.

### 1.4 Ethical consideration

Ethical clearance was not sought for this study since the dataset is freely available in the public domain. However, before the commencement of the survey, ethical approval was obtained from the WHO, CDC, and MTSU. Written informed consent was obtained from the students aged 18 years and above whilst both written parental/guardian consent and child assent forms were sought from those aged 10 to 17 before inclusion into the study.

### 1.5 Results

#### 1.5.1 Prevalence of interpersonal violence among in-school adolescents

The overall prevalence of interpersonal violence among in-school adolescents in SSA was 53.7% of which the prevalence of physical fighting and physical attacked were 29.2% and 43.2% respectively. At the country level, the highest prevalence of interpersonal violence was 64.2% in Liberia with the lowest in Mauritius (32.5%) (see Fig. 1).

#### 1.5.2 Distribution of interpersonal violence across explanatory variables

Table 1 presents the results on the bivariate analysis of proportions of interpersonal violence among in-school adolescents in SSA. The prevalence of interpersonal violence was higher (60.1%) among adolescents aged 14 years or younger compared with those aged 15+ (49.3%). Male adolescents recorded a higher proportion of interpersonal violence compared to their female counterparts. Furthermore, there were higher proportions of interpersonal violence among in-school adolescents who felt hungry (53.9%), had injury (67.7%), were bullied (70.1%), experienced anxiety (61.8%), felt lonely (59.7%), had suicidal ideation (63.2%), had close friends (54.1%), were truant (65.1%), and had no peer support (55.8%) compared to their counterparts. The results showed a higher proportion of interpersonal violence among in-school adolescents who currently consumed alcohol (57.4%), smoked cigarette (73.6%), used marijuana (83.2%), and used tobacco (78.0%). In-school adolescents who reported parent or guardian supervision had a higher proportion of interpersonal violence (54.4%) compared to those who did not have parent or guardian supervision (53.0%).

Also, in-school adolescents who had no connectedness (55.2%), no bonding (55.4%), and no respect for privacy (60.5%) from their parent or guardian had higher proportion of interpersonal violence compared to their other counterparts.

#### 1.5.3 Predictors of interpersonal violence among in-school adolescents in sub-Saharan Africa

Table 2 shows results of the multivariable binomial regression analysis of predictors of interpersonal violence among in-school adolescents in SSA. The odds of interpersonal violence were higher among adolescent who were bullied compared to those who were not bullied (aOR = 2.52, 95% CI = 2.23–2.85). In-school adolescents who had an injury were approximately two times more likely to experience interpersonal violence compared to those who did not have an injury (aOR = 2.42, 95% CI = 2.15–2.72). The odds of interpersonal violence were higher among adolescents who had suicidal attempts compared to those who did not have suicidal attempts (aOR = 1.40, 95% CI = 1.16–1.70). In-school adolescents who were truant were more likely to experience interpersonal violence compared to those who were not truant (aOR = 1.51, 95% CI = 1.33–1.72). Higher odds of interpersonal violence was found among in-school adolescents who used alcohol (aOR = 1.49, 95% CI = 1.06–2.11) and tobacco (aOR = 1.46, 95% CI = 1.23–1.74) compared to those who did not use alcohol and tobacco. In-school adolescents with peer support, parents or guardians bonding, those whose parents or guardians respected their privacy and those aged 15 years or older were less likely to experience interpersonal violence.

**Table 2:** Multivariable binomial regression analysis of predictors of interpersonal violence among in-school adolescents in SSA.

Variables	Physical fighting aOR [95% CI]	Physically attacked aOR [95% CI]	Interpersonal violence aOR [95% CI]
Age group			
14 years or younger	1 [1.00,1.00]	1 [1.00,1.00]	1 [1.00,1.00]
15 years or older	0.50*** [0.44,0.57]	1.01 [0.89,1.14]	0.77*** [0.68,0.88]
**Sex**			
Female	1 [1.00,1.00]	1 [1.00,1.00]	1 [1.00,1.00]
Male	1.11 [0.98,1.26]	1.08 [0.97,1.22]	1.10 [0.98,1.23]
**Felt hungry**			
No	1 [1.00,1.00]	1 [1.00,1.00]	1 [1.00,1.00]
Yes	0.89 [0.73,1.09]	1.08 [0.90,1.30]	1.01 [0.84,1.22]
**Bullied**			
No	1 [1.00,1.00]	1 [1.00,1.00]	1 [1.00,1.00]
Yes	2.30*** [2.02,2.62]	2.16*** [1.90,2.45]	2.52*** [2.23,2.85]
**Injury**			
No	1 [1.00,1.00]	1 [1.00,1.00]	1 [1.00,1.00]
Yes	2.37*** [2.08,2.69]	2.23*** [1.97,2.51]	2.42*** [2.15,2.72]
**Felt anxious**			
No	1 [1.00,1.00]	1 [1.00,1.00]	1 [1.00,1.00]
Yes	1.01 [0.84,1.23]	1.30** [1.08,1.58]	1.20 [1.00,1.45]
**Felt lonely**			
No	1 [1.00,1.00]	1 [1.00,1.00]	1 [1.00,1.00]
Yes	0.97 [0.80,1.17]	1.04 [0.87,1.25]	0.99 [0.82,1.19]
**Suicidal ideation**			
No	1 [1.00,1.00]	1 [1.00,1.00]	1 [1.00,1.00]
Yes	1.24* [1.01,1.52]	1.05 [0.87,1.27]	1.08 [0.89,1.31]
**Suicidal plan**			
No	1 [1.00,1.00]	1 [1.00,1.00]	1 [1.00,1.00]
Yes	1.01 [0.83,1.24]	1.06 [0.88,1.28]	1.05 [0.86,1.27]
**Suicidal attempt**			
No	1 [1.00,1.00]	1 [1.00,1.00]	1 [1.00,1.00]
Yes	1.26* [1.04,1.53]	1.46*** [1.21,1.76]	1.40*** [1.16,1.70]
**Truant**			
No	1 [1.00,1.00]	1 [1.00,1.00]	1 [1.00,1.00]
Yes	1.61*** [1.41,1.84]	1.37*** [1.21,1.56]	1.51*** [1.33,1.71]
**Current cigarette smoking**			
No	1 [1.00,1.00]	1 [1.00,1.00]	1 [1.00,1.00]
Yes	1.85*** [1.31,2.62]	0.98 [0.68,1.41]	1.12 [0.77,1.63]
**Current tobacco use**			
No	1 [1.00,1.00]	1 [1.00,1.00]	1 [1.00,1.00]
Yes	1.75*** [1.25,2.44]	1.60** [1.14,2.26]	1.49* [1.06,2.11]
**Current alcohol use**			
No	1 [1.00,1.00]	1 [1.00,1.00]	1 [1.00,1.00]
Yes	1.49*** [1.25,1.77]	1.30** [1.09,1.56]	1.46*** [1.23,1.74]
**Current marijuana use**			
No	1 [1.00,1.00]	1 [1.00,1.00]	1 [1.00,1.00]
Yes	1.06 [0.68,1.65]	0.94 [0.59,1.49]	1.43 [0.89,2.29]
**Peer support**			
No	1 [1.00,1.00]	1 [1.00,1.00]	1 [1.00,1.00]
Yes	0.74*** [0.65,0.85]	0.93 [0.83,1.06]	0.82** [0.72,0.92]
**Parent or guardian supervision**			
No	1 [1.00,1.00]	1 [1.00,1.00]	1 [1.00,1.00]
Yes	1.11 [0.96,1.27]	1.09 [0.96,1.24]	1.09 [0.96,1.24]
**Parent or guardian connectedness**			
No	1 [1.00,1.00]	1 [1.00,1.00]	1 [1.00,1.00]
Yes	1.00 [0.87,1.15]	1.00 [0.88,1.14]	1.02 [0.90,1.15]
**Parent or guardian bonding**			
No	1 [1.00,1.00]	1 [1.00,1.00]	1 [1.00,1.00]
Yes	0.81** [0.71,0.94]	0.95 [0.83,1.08]	0.87* [0.77,0.99]
**Parent or guardian respect for privacy**			
No	1 [1.00,1.00]	1 [1.00,1.00]	1 [1.00,1.00]
Yes	0.80** [0.70,0.92]	0.88 [0.78,1.00]	0.79*** [0.70,0.90]
**Country**			
Benin	1 [1.00,1.00]	1 [1.00,1.00]	1 1.00,1.00]
Ghana	1.65*** [1.36,2.01]	2.41*** [1.99,2.92]	2.23*** [1.87,2.68]
Liberia	1.74*** [1.39,2.17]	4.00*** [3.22,4.96]	3.13*** [2.54,3.86]
Mauritius	1.20 [0.98,1.49]	1.24* [1.00,1.54]	1.34** [1.11,1.62]
Mozambique	1.26 [0.98,1.63]	1.92*** [1.51,2.44]	1.65*** [1.32,2.06]
Namibia	1.20* [1.00,1.45]	2.11*** [1.77,2.52]	1.63*** [1.38,1.92]
Seychelles	0.91 [0.73,1.12]	1.26* [1.02,1.56]	1.23* [1.01,1.49]
Tanzania	1.41** [1.14,1.73]	7.50*** [6.09,9.23]	5.26*** [4.34,6.38]
*N*	14967	14967	14967
pseudo *R*^2^	0.146	0.117	0.132

Exponentiated coefficients; 95% confidence intervals in brackets; *p < 0.05, **p < 0.01, ***p < 0.001.

### 1.6 Discussion

This study examined the prevalence and factors associated with interpersonal violence among in-school adolescents in SSA. The prevalence of interpersonal violence among in-school adolescents in SSA was found to be 53.7%. The current prevalence is relatively lower than the prevalence reported by a previous study in Ghana (55.7%) (Aboagye et al., 2021); however, this present study showed a higher prevalence than studies conducted in Sri Lankan (Senanayake et al., 2019) and Tanzania (Peltzer & Pengpid, 2014) which recorded prevalence rates of 44.2% and 53.1% respectively. Liberia had the highest prevalence(64.2%) of interpersonal violence while Mauritius had the lowest prevalence (32.5%). The establishment of a strategy and policy aimed at reducing violent behaviour among Mauritius' school-aged adolescents could be one of the reasons for the lower prevalence of interpersonal violence among in-school adolescents in the country (Abiodun et al., 2021).

In this study, male adolescents (53.7%) recorded a higher proportion of interpersonal violence compared to their female counterparts (53.6%); nevertheless, there was no significant difference in their reporting of interpersonal violence, a finding which corroborates with previous studies (Aboagye et al., 2021; Swan et al., 2013).

Adolescents who had injuries were also significantly more likely to experience interpersonal violence in SSA. This result is consistent with previous studies conducted in Ghana (Aboagye et al., 2021), Tanzania (Peltzer & Pengpid, 2014), and Southeast Asia (Rudatsikira et al., 2007). A study by Acquah, Wilson, and Doku (2014) reported that being injured was associated with a higher chance of youth violence (e.g., being physically attacked). The plausible reason could be that injured adolescents who may experience scorns, mockery, and taunts from peers may retaliate in the form of physical attacks (Murgayroyd et al., 2015). This finding supports the individual level factors of the ecological framework of interpersonal violence and affirm how previous experience of violence (injuries) predispose an individual to interpersonal violence (WHO, 2021).

Truancy was significantly associated with interpersonal violence among in-school adolescents in SSA. Truant adolescents were more likely to experience interpersonal violence. This finding confirms findings of previous studies (Senanayake et al., 2019; Peltzer & Pengpid, 2014; Lee et al., 2007). It is worth indicating that adolescents who miss school or skip classes have a higher probability of finding themselves in violent confrontations with other students. Also, in-school adolescents involved in violence are more likely to be expelled or drop out (Shireen et al., 2014; Sharma et al., 2008). Evidence from literature has proven that truant students are capable of demonstrating different kinds of violent and risky behaviours (Ramberg et al., 2019; Senanayake et al., 2019; Kelly et al., 2015; Kipping et al., 2012; Yen et al., 2010). Truants in school are more likely to be victims of interpersonal violence, according to previous study (Aboagye et al., 2021) done in Ghana. The explanation for this could be that students who are absent from school have a high level of resistance to aggressive behaviour, making them more vulnerable to getting involved in interpersonal violence. The finding that truancy is associated with interpersonal violence aligns with the individual level factors of the ecological framework of interpersonal violence and affirm how truancy predisposes an individual to interpersonal violence (WHO, 2021).

In line with previous studies (Aboagye et al., 2021; Peltzer & Pengpid, 2014), we found that drug use such as tobacco use and current alcohol use were significantly associated with interpersonal violence among in-school adolescents in SSA. Evidence from literature has shown that substance use, specifically tobacco use and alcohol play a significant role in irresponsible behaviours in school settings and the society (Aboagye et al., 2021; Pengpid & Peltzer, 2019). In-school adolescents who consumed alcohol and smoked tobacco during the study were significantly more likely to experience interpersonal violence compared to those who did not consume alcohol and smoke tobacco. Considering that adolescents who consume alcohol and smoke tobacco may engage in delinquent behaviours, they are also likely to become involved in an altercation in which they do not know the cause. In-school adolescents who consume/smoke alcohol and tobacco are at risk of engaging in multiple risk behaviours, including interpersonal violence because it distorts cognition by impairing one's sense of judgement and decision making, which usually lead to negative action tendencies (Beyene et al., 2019; Maynard et al., 2016; Pickett et al., 2013). The finding that drug and alcohol use is a risk factor for interpersonal violence is in line with the individual level factors of the ecological framework of interpersonal violence and affirm how drug and alcohol use predisposes an individual to interpersonal violence (WHO, 2021).

Bullying victimization was significantly associated with interpersonal violence among in-school adolescents in SSA. The study found that in-school adolescents who were bullied were significantly associated with interpersonal violence. This result is consistent with previous studies conducted elsewhere (Cecen-Celik & Keith, 2019; Peltzer & Pengpid, 2014; Pickett et al., 2013). These studies have shown that bullying victimization was associated with increased violent behaviour in adolescents. Adolescents who are victims of bullying are more likely to use the same violent behaviour to defend themselves and cope with conflict. This finding is congruent with the co-occurrence model of interpersonal violence, which posits that, individuals who experience at least one form of violence are more likely to experience interpersonal violence (Hamby et al., 2012). However, finding ways of knowing students who are often bullied by colleague students may help to reduce the incidence of violence at school. Therefore, anti-bullying school interventions are required in school settings.

Suicidal attempt was also significantly associated with interpersonal violence among in-school adolescents. The study reported that in-school adolescents in SSA who attempted suicide were more likely to engage in interpersonal violence. Studies conducted in different settings have shown the association between maladaptive behaviours (e.g., drug use, suicidal attempts) and physical violence through negative feelings of diminished self-worth (Pengpid & Peltzer, 2020; Radiff et al., 2016); therefore, in-school adolescents who have been physically attacked in any form of violence may experience low self-esteem and may develop negative thoughts about their personality, thus show dysfunctional behaviours such as suicidal attempts (Tsaousis, 2016). To manage the issue of suicide attempts and interpersonal violence, heads and management of schools need to develop suicide preventive interventions for school students considering prioritising their experience of bullying and substance use.

In-school adolescents with peer support, parents or guardians bonding and those whose parents or guardians respected their privacy were less likely to experience interpersonal violence. Similar finding was obtained in a previous study (Aboagye et al., 2021). It is likely that in-school adolescents who have received peer support and bonding time with their parents or guardians acquire cognitions and sentiments of belongingness that protect them against interpersonal violence. Adolescents who receive peer support at school also feel socially safe from any type of violence (Aboagye et al., 2021). A similar finding has been reported by Randall et al. (2014). The possible reason could be that in-school adolescents may have the parental support structure and be free from peer victimization, emotional and mental stress. Poor parenting may cause physical and emotional stress as well as impaired social and cognitive development (Waldvogel et al., 2008; Wagner et al., 2003). This finding affirms the role of family members, friends and peers in interpersonal violence. The finding shows that the support individuals receive through their interactions serves as protective factor against interpersonal violence, as evidenced in the ecological framework of interpersonal violence (WHO, 2021). Preventive interventions on interpersonal violence should be put in place to encourage peer relationships. To increase acceptance and belongingness among in-school adolescents, school officials should expand interventions such as social support networks through supervision and monitoring. Concerning age, we found that there was an association between age group and interpersonal violence. The study revealed that in-school adolescents aged 15 years or older were less likely to experience interpersonal violence compared to those aged 14 years or younger. The plausible explanation could be that adolescents aged 15 years or older may have control over their physical aggression survivorship and perpetration. Also, a possible reason could be that in-school adolescents aged 15 years or older may have developed problem-solving skills and as such, they are less likely to consider committing violence than the younger ones (Muula et al., 2007). This finding is consistent with previous studies conducted in different settings (Pengpid & Peltzer, 2020; Peltzer & Pengpid, 2019). The finding that age is associated with interpersonal violence aligns with the important role of socio-demographic characteristics in interpersonal violence victimization, as explained in the ecological framework for interpersonal violence (WHO, 2021). Preventive interpersonal violence interventions with adolescent school learners should seek to address their perpetration.

### 1.7 Policy and practice implications

Our findings have some policy and practice implications. Our findings highlight the need to implement and strengthen already existing programmes and intervention that target in-school adolescents especially truant adolescents, adolescents who drink alcohol, those who smoke cigarettes, and those who were bullied on interpersonal violence prevention. In an attempt to curb interpersonal violence among in-school adolescents in SSA, school authorities and policymakers must design and implement proactive intervention such as peer educator network system, face-to-face counseling sessions, substance use cessation therapy, and Rational Emotive Behavioural Education (REBE) among in-school children to prevent them from engaging in risky behaviors, including physical fights.

### 1.8 Strengths and limitations

The main strength of the study is the use of nationally representative datasets of the various countries considered in the study. This is significant, given that it allows for the generalizability of the findings in the selected countries. The study also used sophisticated data analysis tools that ensured rigorous analysis of the data. Aside from these, the data collection featured well-experienced field assistants and well-designed questionnaires which resulted in a higher response rate. This guarantees the reliability of the findings. Despite these strengths, the study also has some limitations that need to be acknowledged. The first limitation concerns the research approach adopted. In this regard, it is important to note that the cross-sectional study design, this study could only show factors associated with interpersonal violence among in-school adolescents in SSA, and not establish causal relationships. Since the in-school adolescents filled out the survey questionnaire themselves, it might have led to incorrect responses. In-school adolescents who were absent on the day of data collection were not included. The study might have encountered issues of recall bias, leading to under- and over-reporting of interpersonal violence and other variables.

## 2 Conclusion

This study has offered insights into interpersonal violence among in-school adolescents in sub-Saharan Africa. More than half (53.7%) of in-school adolescents included in this study experienced interpersonal violence. Age group, current alcohol use, injury, bullying victimization, suicide attempt, peer support, feeling anxiety, parent or guardian respect for privacy, current cigarette smoking, current tobacco use, and truancy were found to be the factors associated with interpersonal violence among in-school adolescents in sub-Saharan Africa. These factors need to be considered when designing interventions aimed at preventing interpersonal violence in schools.

## Funding

The study did not receive any funding.

## Declaration of competing interest

The authors declare that they have no competing interests.
