# Supplementary material for: Interpersonal violence among in-school adolescents in sub-Saharan Africa: Assessing the prevalence and predictors from the Global School-based health survey
Source: SSM Popul Health. 2021 Oct 1;16:100929. doi: 10.1016/j.ssmph.2021.100929 (PMC8502764; doi:10.1016/j.ssmph.2021.100929)
Supplement: Multimedia component 1 [file mmc1.docx]

**Table S1: Links to questionnaires and Datasets**

| Country | Link to dataset | Link to questionnaire |
| --- | --- | --- |
| 1. Benin(2016) | https://www.who.int/ncds/surveillance/gshs/BJH2016_GSHS_Data_public_use.dat (accessed on 1/4/2021) | <https://www.who.int/ncds/surveillance/gshs/BJH2016_GSHS_Data_public_use_codebook.pdf?ua=1> (accessed on 1/4/2021) |
| 1. Ghana(2012) | <https://www.who.int/chp/gshs/GHDH2012_public_use.dat> (accessed on 1/4/2021) | <https://www.who.int/chp/gshs/2012_Ghana_GSHS_Questionnaire.pdf?ua=1>(accessed on 1/4/2021) |
| 1. Liberia(2017) | <https://extranet.who.int/ncdsmicrodata/index.php/catalog/646/download/4628> (accessed on 1/4/2021) | <https://extranet.who.int/ncdsmicrodata/index.php/catalog/646/download/4624> (accessed on 1/4/2021) |
| 1. Mauritius (2017) | <https://extranet.who.int/ncdsmicrodata/index.php/catalog/669/download/4823> (accessed on 1/4/2021) | <https://extranet.who.int/ncdsmicrodata/index.php/catalog/669/download/4819> (accessed on 1/4/2021) |
| 1. Mozambique(2015) | <https://www.who.int/chp/gshs/MZH2015_public_use.dat> (accessed on 1/4/2021) | <https://www.who.int/chp/gshs/MZH2015_public_use_codebook.pdf?ua=1> (accessed on 1/4/2021) |
| 1. Namibia(2013) | <https://www.who.int/chp/gshs/NBH2013_public_use.dat> (accessed on 1/4/2021) | <https://www.who.int/chp/gshs/NBH2013_public_use_codebook.pdf?ua=1> (accessed on 1/4/2021) |
| 1. Seychelles(2015) | <https://www.who.int/chp/gshs/SHH2015_public_use.dat> (accessed on 1/4/2021) | <https://www.who.int/chp/gshs/SHH2015_public_use_codebook.pdf?ua=1> (accessed on 1/4/2021) |
| 1. Tanzania(2014) | <https://www.who.int/chp/gshs/TZH2014_public_use.dat> (accessed on 1/4/2021) | <https://www.who.int/chp/gshs/TZH2014_public_use_codebook.pdf?ua=1> (accessed on 1/4/2021) |
