# Supplementary material for: Interpersonal violence among in-school adolescents in sub-Saharan Africa: Assessing the prevalence and predictors from the Global School-based health survey
Source: SSM Popul Health. 2021 Oct 1;16:100929. doi: 10.1016/j.ssmph.2021.100929 (PMC8502764; doi:10.1016/j.ssmph.2021.100929)
Supplement: Multimedia component 2 [file mmc2.docx]

**Table S1. ﻿ Study variables**

| **Variables** | **Question** | **Response options and recoding** |
| --- | --- | --- |
| **Outcome variable** | | |
| Fight | ﻿During the past 12 months, how many times were you in a physical fight? | ﻿﻿1 = 0 times; to 8 = 12 or more times (coded as 1 = No; and 2–8 = Yes) |
| Attacked | ﻿During the past 12 months, how many times were you physically attacked? | 1=0 days,2= 1or 2 days, 3=3 to 5 days, 4=6 to 9 days, 5= 10 or more (coded as 1=No; and 2-8=Yes) |
| **Explanatory variables** | | |
| Socio-demographic characteristics | | |
| Age | How old are you? | 1=12, 2=13, 3=14, 4=15, 5=16, 6=17, 7=18 years (coded as 0=12-14, 15-19) |
| Sex | What is your sex? | 1=male, 2=female  (coded 2=Female, 1=male) |
| Hunger (proxy of socioeconomic status) | Went hungry past 30 days | 1=never, 2=Rarely, 3=sometimes, 4=most of the times, 5=always  (coded 1-3=No; and 4-5=Yes) |
| Psychosocial environmental factors | | |
| Tobacco use | ﻿During the past 30 days, on how many days did you use any other form of tobacco, such as chewing tobacco leaves? | 1 = 0 days; to 7 = All 30 days  (coded as 1 = No; and 2–7 = Yes) |
| Alcohol use | ﻿During the past 30 days, on how many days did you have at least one drink containing alcohol? | ﻿1 = 0 days; to 7 = All 30 days  (coded as 1 = No; and 2–7 = Yes) |
| Cigarette smoking | ﻿During the past 30 days, how many days did you smoke cigarette? | 1 = 0 days; to 7 = All 30 days  (coded as 1 = No; and 2–7 = Yes) |
| Marijuana use | During the past 30 days, how many times have you used marijuana (also called wee, Jah, Indian hemp, ahabammono, and ganja) | 1=0 times; to 5=20 or more times (coded as 1=No; and 2-5=Yes) |
| Anxiety | ﻿During the past 12 months, how often have you been so worried about something that you could not sleep at night? | ﻿1 = never to 5 = always  (coded 1 - 3 = No; and 4 – 5 = Yes**)** |
| Loneliness | ﻿During the past 12 months, how often have you felt lonely? | ﻿1=never, 2=rarely, 3= sometimes, 4 = most of the time to 5 = always (coded as 1-3 = No; and 4- 5 = Yes) |
| Injury | ﻿During the past 12 months, how many times were you seriously injured? | 1 = 0 times; to 8 = 12 or more time (coded as 1 = No; and 2–8 = Yes) |
| Truancy | ﻿During the past 30 days, on how many days did you miss classes or school without permission? | 1=0 days,2= 1or 2 days, 3=3 to 5 days, 4=6 to 9 days, 5= 10 or more  (coded as 1=No; and 2-5=Yes) |
| Suicidal ideation | During the past 12 months, did you ever seriously consider attempting suicide?” | 1 = yes, 2 = no  (coded 2 = No; and 1 = Yes) |
| Suicide plan | During the past 12 months, did you make a plan  about how you would attempt suicide? | 1 = yes, 2 = no  (coded 2 = No; and 1 = Yes) |
| Suicidal attempt | During the past 12 months, how many times did you actually attempt suicide? | 1=0 times; to 5= 6 or more times (coded as 1=No; and 2-5=Yes) |
| Bullied | ﻿During the past 30 days, how were you bullied most often? | ﻿1 = 0 times; to 8 = 12 or more times (coded as 1 = No; and 2–7 = Yes) |
| Close friends | ﻿How many close friends do you have? | 1=0 to 4=3 or more  (coded as 1=No; and 2-4=Yes) |
| Helpful  (Peer support) | ﻿During the past 30 days, how often were most of the students in your school kind and helpful? | 1=never, 2=Rarely, 3=sometimes, 4=most of the times, 5=always  (coded as 1-3 = No; and 4–5 = Yes) |
| Parents check homework (parental supervision) | ﻿During the past 30 days, how often did your parents or guardians check to see if your homework was done? | 1=never, 2=Rarely, 3=sometimes, 4=most of the times, 5=always  (coded as 1-3 = No; and 4–5 = Yes) |
| Understand problems  ﻿ (Parental  Connectedness | ﻿During the past 30 days, how often did your parents or guardians understand your problems and worries? | ﻿1=never, 2=Rarely, 3=sometimes, 4=most of the times, 5=always  (coded as 1-3 = No; and 4–5 = Yes) |
| Know what adolescent do free time (﻿Parental or guardian  Bonding) | ﻿During the past 30 days, how often did your parents or guardians really know what you were doing with your free time? | ﻿1=never, 2=Rarely, 3=sometimes, 4=most of the times, 5=always  (coded as 1-3= No; and 4–5 = Yes) |
| Parental or guardian respect for Privacy | During the past 30 days, how often did your parents or guardians go through your things without your approval? | 1=never, 2=Rarely, 3=sometimes, 4=most of the times, 5=always  (coded as 1-3 = No; and 4–5 = Yes) |
